# P87 Driving behaviour change in antimicrobial prescription indication documentation through personalized feedback

**DOI:** 10.1093/jacamr/dlaf230.094

**Published:** 2025-12-04

**Authors:** Nicola Jones, Bee Yean Ng, Caroline Dean, Louise Dunsmure

**Affiliations:** Department of Pharmacy, Oxford University Hospitals NHS Foundation Trust, Oxford, UK; Department of Infectious Diseases, Oxford University Hospitals NHS Foundation Trust, Oxford, UK; Department of Pharmacy, Oxford University Hospitals NHS Foundation Trust, Oxford, UK; Department of Pharmacy, Oxford University Hospitals NHS Foundation Trust, Oxford, UK; Department of Pharmacy, Oxford University Hospitals NHS Foundation Trust, Oxford, UK

## Abstract

**Background:**

Documentation of indication for antimicrobial prescription is a cornerstone of antimicrobial stewardship (AMS). At our UK NHS Trust, the e-prescribing system for antimicrobials mandates an indication be completed. This is currently free text and quality can be variable. There are occasions where rather than recording a meaningful indication, ‘nonsense fields’ e.g. a full stop, a number, bleep number or dash were used presumably as the prescriber perceives this as a quick way to ‘get past’ a mandatory field. The antimicrobial stewardship team implemented a project over a 3 year period to monitor completion of the indication field and to feedback to prescribers.

**Objectives:**

To motivate the individual to change their behaviour and to reduce the number of inappropriate indications to improve stewardship.

**Methods:**

A report was built using the Electronic Prescribing System on a monthly basis extracts all antimicrobial prescriptions within the previous month and is used to identify inappropriate indications. The AMS audit assistant identified inappropriate indications and emailed the prescriber to feedback that the indication documentation was inappropriate. The content of the email was individualized with specifics of the prescription using an agreed template to ensure personalized feedback. Any prescribers repeatedly documenting inappropriate indications were contacted by the AMS lead to understand reasons for incorrectly prescribing antimicrobials. The importance of correctly prescribing antimicrobials was emphasized.

**Results:**

In total 336 377 antimicrobial prescriptions were reviewed over a 3 year period and of those, 1087 (0.32%) inappropriate indications were identified requiring email feedback. As a result of feedback, the number of inappropriate indications has decreased over the 3 years. In Year 1, 0.4% (531/131 115) indications were inappropriately documented. In Year 2, 0.28% (319/114 471) indications were inappropriately documented. In Year 3, 0.26% (237/90 791) indications were inappropriately documented. This shows a downward trend of inappropriately documented indications hence quality of antimicrobial prescribing has improved. This downward trend also demonstrates a change in behaviour amongst prescribers

**Conclusions:**

This project has led to an improvement in antimicrobial prescribing practices, with prescribers becoming more aware of the importance of accurate documentation and showing increased vigilance. Reminders to clearly state indications not only enhanced clinical decision-making but also prompt prescriber to reassess the appropriateness of antimicrobial use, aligning with the principles of ‘start smart then focus’. Antimicrobial stewardship pharmacists reported that improved accuracy in prescribing indications has made it easier to audit antimicrobial prescribing practices. This improved data quality, enabling data-driven interventions and enhancing accountability that inform future antimicrobial stewardship strategies and interventions to counteract antimicrobial resistance. Additionally, a notable reduction in the completion of irrelevant or vague indication fields demonstrates the project’s impact. This reinforces prescribers' awareness that their entries are being monitored, thereby encouraging accuracy. Future efforts will focus on integrating automated, timely messages via electronic records and developing a drop-down menu for indication selection.

